# Prevalence of Panton-Valentine Leukocidin Gene among Community Acquired* Staphylococcus aureus*: A Real-Time PCR Study

**DOI:** 10.1155/2018/4518541

**Published:** 2018-09-02

**Authors:** Amit Karmakar, Debarati Jana, Kunal Dutta, Parimal Dua, Chandradipa Ghosh

**Affiliations:** Microbiology Laboratory, Department of Human Physiology with Community Health, Vidyasagar University, Paschim Medinipur, West Bengal 721102, India

## Abstract

Panton-Valentine leukocidin (*luk-pv*) is a cytotoxin that causes leukocyte destruction and tissue necrosis. The aim of this study was to determine the prevalence of the* pv1*,* mecA*, and* nuc* genes in* Staphylococcus aureus* isolates obtained from anterior nares and superficial infection sites of skin in a slum population of West Bengal, India. Expression level of* pv1* gene was also analysed. Twenty-two* S. aureus *strains were isolated, and phenotype and genotype specific examinations for* S. aureus* isolates were carried out. Molecular identification was done by PCR using species-specific 16S rRNA primer pairs and finally 22 isolates were found to be positive as* S. aureus*. The antibiotic responsiveness of all these isolates and the minimum inhibitory concentration (MIC) of MRSA isolates were determined using the broth dilution method with vancomycin. Antibiogram analysis of isolated* S. aureus *strains with respect to different antimicrobial agents revealed antibiotic resistance ranging from 27 to 91%. The results of MIC for vancomycin showed 95% of strains to be VSSA and 5% to be VISA. 68% isolates were resistant to methicillin. All the isolates were subjected to detection of* pv1, mec*A, and* nuc* genes, and 9%, 68%, and 27% were found to harbour* pvl, mec*A, and* nuc* genes, respectively. All the MRSA strains produced high to moderate levels of biofilm.* pvl* gene expression was carried out* in vitro* by Real-Time PCR. The low ∆Ct value (0.493) was indicative of high expression of* pvl* in one* S. aureus *strain. Thus, detection of* pvl *gene in community acquired* S. aureus* indicates the emergence of pathogenic* S. aureus* in community setup in the studied region. The existing exploration is extremely imperative and informative for the high level multi-drug resistant* S. aureus *infections inclusive of MRSA.

## 1. Introduction

To date, the major human pathogen,* Staphylococcus aureus, *has become a threat to our lives, because of elaboration of several different virulence factors. Panton-Valentine leukocidin (PVL) is one of the most important virulence factors of* S. aureus*. This beta pore forming cytotoxin is associated with tissue necrosis and also causes disruption of leukocyte membranes [1]. The genetic material of bacteriophage has great contribution for producing PVL cytotoxin. PVL carrying* S. aureus *strains are more virulent and highly transmissible strains than PVL negative* S. aureus*.* pvl *gene that encodes PVL cytotoxin comprises two exoprotein subunits, encoded by LukS-PV and LukF-PV [2, 3]. These two co-transcribed genes act together as a subunit to form a pore by assembling in the cell membranes of host immune cells particularly the white blood cells, monocytes, and macrophages [4]. PVL carrying* S. aureus* is responsible for different life-threatening invasive diseases, and also skin and soft tissue infections. PVL-SA infected skin is red and inflamed with pus. It can have different other appearances like cellulitis, abscesses, boils, folliculitis, etc. At first, PVL carrying* S. aureus *infects skin and soft tissues but the infection gradually spreads to the lung and disrupts the lung tissues, causing hemorrhagic necrotizing pneumonia, one of the most lethal diseases caused by* S. aureus *[5].

In recent time, there have been an overall increase in the prevalence of* pvl* positive* S. aureus *worldwide. But variable rates of prevalence have been reported from different countries, i.e., 12.8% in China [6], 30% in Germany [7], 45.3% in Japan, and most remarkably 97% in USA [8].

**Figure 1:**
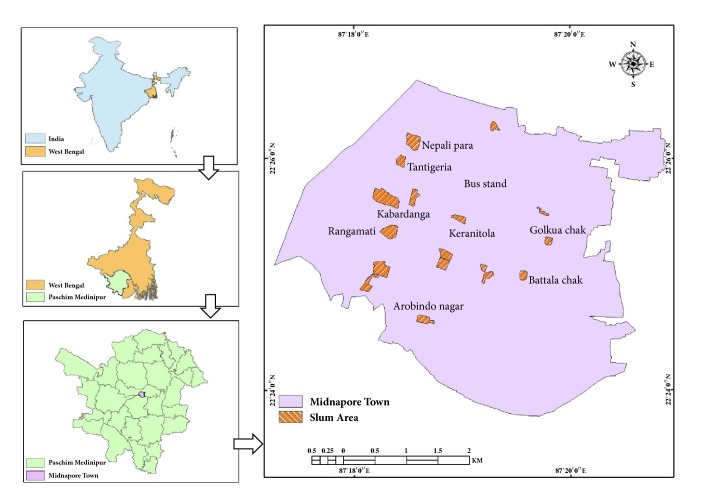
Study region including different slum areas in Midnapore town (Lat. 22° 23′41.57 N to 22° 26′22.02^″^ N; Long. 87°17′23.65^″^ E to 87° 20′ 15.35^″^ E).

To date PVL has become most important and significant virulence factor of community acquired* S. aureus*. The prevalence of* pvl* genes among MRSA isolates has not been adequately reported from India. This study was undertaken to investigate the PVL prevalence and* pvl* expression level along with certain other virulent markers from state of West Bengal, India. The study area is demonstrated in Figure 1.

## 2. Materials and Methods

### 2.1. Sample Collection

A total of 25 non-repeat community strains of* S. aureus *were collected from human subjects of different slum areas at Midnapore town (Lat. 22°23′41.57^″^ N to 22°26′22.02^″^ N; Long. 87°17′23.65^″^ E to 87°20′15.35^″^ E) in the state of West Bengal, India, from November 2015 to March 2016. The protocols in present study with human subjects got ethical clearance from Institutional Review Board (IRV) who follows the Indian Council of Medical Research (ICMR) ethical guidelines for biomedical research on human subjects. Bacterial isolates were categorised into three groups according to the site of collection, age group, and sex (Table 1). For sample collection, sterile swabs with rayon tips were used; for moist wound it was used directly but for dry wound it was moistened with sterile saline so that it could increase the chance of recovery of the organism from the infected sites.

### 2.2. Isolation and Identification of* S. aureus*

All the samples were transferred into 2 gm% Luria broth (one for each specimen) and incubated at 37°C in shaking incubator. After 16-18 hours all the samples were inoculated onto mannitol salt agar plate and incubated for 24-25 hours at 37°C [14]. All yellow pigmented colonies were inoculated into LB agar for culture preservation at 4°C. The resulting growth from respective plates of media was examined for colony characteristic and morphology. Each culture underwent Gram staining and was tested for production of catalase, free coagulase, yellow pigment, and thermonuclease (TNase) according to method described by Lancette and Tatini [15].

### 2.3. DNA Extraction from Bacterial Culture Isolates

For extraction of DNA from bacterial culture, the rapid boiling method was followed [14]. In brief, 4 to 5 single colonies from overnight grown culture were inoculated in 200 *μ*l of sterile Milli-Q water and boiled at 99°C for 10 minutes. After centrifugation at 14000 rpm for 10 min, the supernatant was collected in a sterile microcentrifuge tube gently. The resultant template DNA was stored at −20°C and 2 *μ*l of each sample was used for PCR analysis.

### 2.4. Molecular Identification through PCR Amplification of* 16S rRNA* Gene

Phenotypically isolated and confirmed* S. aureus *isolates were further tested by PCR amplification assay using primer pairs (Table 3) for species-specific* 16S rRNA* gene. These primers amplified 228 bp fragment from* 16S rRNA* gene of these isolated* S. aureus* strains. The PCR procedure was carried out in a 25 *μ*l PCR tube and each of the reaction mixtures contained 2.5 *μ*l PCR buffer (10X); 1 *μ*l (1U/*μ*l) of Taq DNA polymerase; 2 *μ*l dNTPs (200 mM each); 1 *μ*l of each primer (10 pmol/*μ*l); 2.5 *μ*l (10 ng/*μ*l) of template DNA; and 15.75 *μ*l PCR grade water. The amplifications took place by the process of PCR thermocycling in a thermal cycler (Eppendorf, Germany) commencing with an initial denaturation at 94°C for 2 minutes followed by 33 cycles, each consisting of denaturation at 94°C for 30 sec, annealing at 50°C for 30 sec, and polymerization at 72°C for 45 sec with a final extension at 72°C for 4 minutes.* S. aureus *ATCC 25923 was used as positive control in this experiment.

### 2.5. Biochemical Characterization of* S. aureus *Strains

The bacterial strains which were confirmed as* S. aureus *by species-specific* 16S rRNA* were further evaluated by different biochemical tests including mannitol fermentation and growth on high salt concentration, gelatin hydrolysis, urea hydrolysis, protease activity on milk agar medium, lipase production on egg yolk agar medium (HiMedia, Mumbai), and hydrolysis of esculin by standard method [16–19] and congo red agar (CRA) test [20].

### 2.6. Biofilm Assay

Biofilm assay was done in borosilicate glass tubes according to the method described by Watnick et al. [21]. Briefly, the isolates were grown in 3 ml of Luria broth overnight. Then 5 *μ*l of the overnight culture was added to 500 *μ*l of sterile LB in test tubes to make a dilution of 1:100. Test tubes were kept standing statically for 24 hrs and after that the test tubes were rinsed vigorously with distilled water to remove all nonadherent cells. Further, 600 *μ*l of 0.1% (w/v) crystal violet was added and incubated for 30 minutes. Test tubes were then rinsed vigorously with distilled water. 1 ml DMSO was added to it followed by vortexing. After incubation for 10 minutes optical density was measured at 570 nm. Each assay was performed in triplicate. The mean values of optical densities obtained were categorised into three classes as suggested by Cha et al. [22], such that the strains with OD_570_< 0.2, 0.2≥ OD_570_≤1.0, and OD_570_> 1.0 were defined as biofilm nonformers and biofilm formers of weak level and strong level, respectively.

### 2.7. Antibiotic Susceptibility Profile of Isolated* S. aureus*

The antibiotic susceptibility pattern of* S. aureus *isolates was done by disk diffusion method using commercial antibiotic disks procured from HiMedia, Mumbai [23]. The antibiotic disks used were as follows: ampicillin (30*μ*g), nalidixic acid (30*μ*g), chloramphenicol (30*μ*g), streptomycin (10*μ*g), kanamycin (30*μ*g), cefoxitin (30*μ*g), novobiocin (30*μ*g), and erythromycin (10*μ*g). Antibiotic susceptibility of those isolates was evaluated according to the Clinical and Laboratory Standard Institute (CLSI) [23]. Minimum Inhibitory Concentration (MIC) of vancomycin was done by the broth dilution method to distinguish VSSA from VRSA according to the Clinical and Laboratory Standard Institute (CLSI, 2015) [24].

### 2.8. Detection of* mecA, nuc, *and* pvl *Gene

Detection of* mecA, nuc*, and* pvl* genes of* S. aureus* was done by PCR amplification assay using gene-specific primer pairs (Table 3). The PCR procedure was carried out in a 25 *μ*l PCR tube and each of the reaction mixtures contained: 2.5 *μ*l PCR buffer (10X); 1 *μ*l (1U/*μ*l) of Taq DNA polymerase; 2 *μ*l dNTPs (200 mM each); 1 *μ*l of each primer (10 pmol/*μ*l); 2.5 *μ*l (10 ng/*μ*l) of template DNA; and 15.75 *μ*l PCR grade water. The amplifications took place by the process of PCR thermocycling in a thermal cycler (Eppendorf, Germany), commencing with an initial denaturation at 94°C for 2 minutes followed by 33 cycles, each comprising 94°C for 1 minute and annealing at 55°C for 45 sec, 50°C for 50 sec, and 50°C for 45 sec for* mecA, nuc, *and* pvl* gene, respectively, and a common extension of 72°C for 45 sec with a final extension at 72°C for 4 minutes. A positive control of ATCC 33591, ATCC 25923, and ATCC 49775* S. aureus *was used, respectively, in each of these experiments. The PCR amplicons were visualized in agarose gel containing ethidium bromide (1 *μ*g/ml) under Gel Doc™XR system (Bio-Rad, USA) and photographs were taken for image analysis. Fragments of DNA 147 bp, 270 bp, and 433 bp corresponded to* mecA*,* nuc*, and* pvl* gene, respectively.

### 2.9. Real-Time Polymerase Chain Reaction of* pvl *Gene

Total RNA was extracted using Pure Link™ RNA Mini Kit (Invitrogen, Carlsbad, CA) and quantified using UV-Vis spectrophotometer (Nano Drop 2000, Thermo Fisher). Total RNA was then converted to cDNA by using Verso cDNA synthesis kit (Thermo Scientific, USA). The standard reaction contained 1x Power SYBR Green PCR master mix (Applied Biosystems), primers pairs (Table 3), and cDNA as template strand. The temperature program for 40 cycles was set to denaturation at 94°C for 1 minute and annealing at 55°C for 30 sec. The reaction was conducted on Step One Plus 96-Well Real-Time PCR System (Applied Biosystems). The samples were analysed in triplicate and* rec*A was used as endogenous control for normalization [25].

### 2.10. Statistical Analysis

Data analysis and plotting of data were done using Microsoft Excel. Mean and standard error of mean was also calculated for each quantitative variable using Microsoft Excel.

## 3. Results

**Figure 2:**
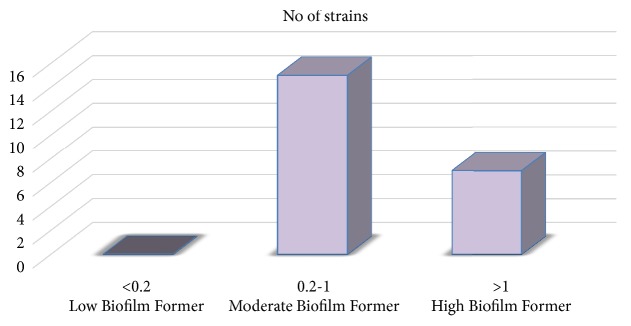
Biofilm formation ability of community strains from slum area.

**Figure 3:**
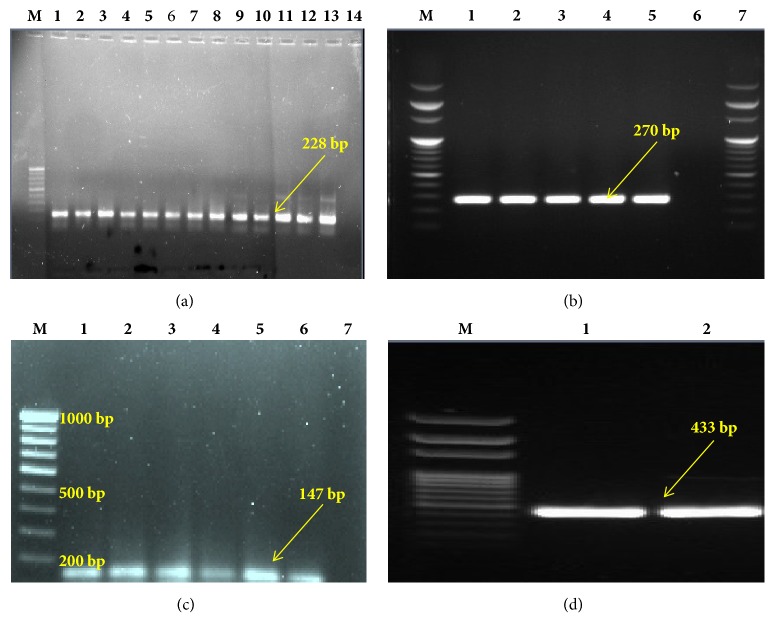
(a) Agarose gel electrophoresis pattern for identification of* Staphylococcus aureus *specific 16S rRNA gene. M, molecular weight marker. Lane 1-13, different clinical strains of* Staphylococcus aureus*. Lane 14, negative control,* Escherichia coli *(SM10 *λ*pir). (b)* nuc *gene. M, DNA molecular weight marker (100 bp DNA ladder); Lane 1, positive control; Lanes 2, 3, 4, and 5, 270 bp band obtained with DNA from clinical strains. Lane 6, negative control (*Staphylococcus epidermidis*). (c) Agarose gel electrophoresis patterns showing PCR-amplified products for the* mecA *gene. M, DNA molecular weight marker (100 bp DNA ladder). Lane 1, positive control; Lanes 2, 3, 4, 5, and 6, 147 bp band obtained with DNA from MRSA clinical strains. Lane 7, negative control (*Staphylococcus epidermidis*). (d) Agarose gel electrophoresis patterns showing PCR-amplified products for the* pvl* gene. Lanes 1 and 2, 433 bp band obtained with DNA from MRSA clinical strains.

**Figure 4:**
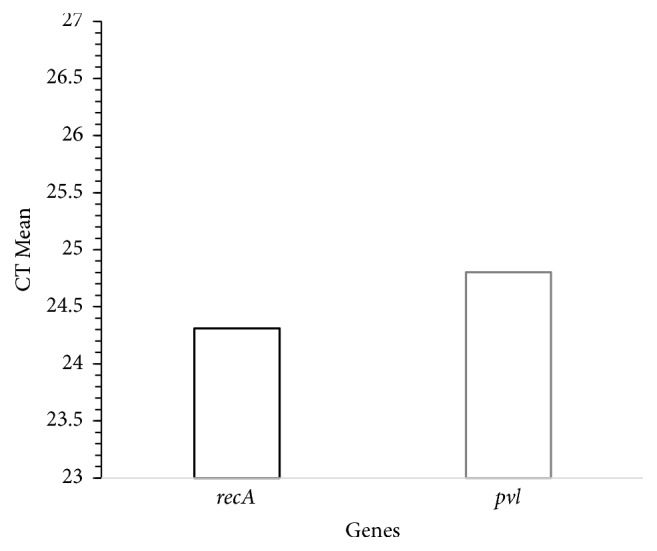
Comparative analysis of the cycle threshold by qRT-PCR assay. Black bar represents the housekeeping gene,* recA,* and gray bar represents* pvl *gene.

A total of 25 nonduplicate isolates of* S. aureus *were obtained from different slum areas in Midnapore town, West Bengal, India, and included in this study. Samples were collected from nasal swab and superficial infection sites of the subjects. Among 25 isolates, 22 strains (88%) were isolated using selective MSA media and then these isolates were identified as* S. aureus *by several phenotypic expressions by means of different biochemical tests (Table 2). All* S. aureus *isolates in this study were confirmed by PCR using the species-specific primer pair (Table 3). In this study, we found that 100%, 91%, and 23% isolates were positive for catalase, coagulase, and heat-stable nuclease, respectively. Of the isolated strains, 68%, 55%, and 68% produced protease, lipase, and nonwhite pigmented colonies, respectively. Rates of resistance of* S. aureus *ranged from 27 to 91% to different antibiotics. Resistance of* S. aureus *isolates to ampicillin (91%), nalidixic acid (91%), chloramphenicol (36%), streptomycin (27%), kanamycin (55%), cefoxitin (68%), novobiocin (64%), and erythromycin (82%) was found.* S. aureus *isolates showing resistance to at least one agent from three or more antimicrobial categories is labeled as multi-drug resistant. Accordingly, all* S. aureus *strains isolated in this study were found to be multi-drug resistant. In the present study, the prevalence rate of MRSA was found to be 68%. It was also found that 5% of the isolated* S. aureus *were vancomycin intermediate and 95% of the isolated* S. aureus* were vancomycin sensitive. None of the strains were found to be VRSA. In general observations, slime-producing strains appear as black colonies, whereas non-slime-producing strains appear as red colonies in CRA plates. Among the community acquired isolates, 15 out of 22 (68%)* S. aureus *strains appeared to be slime-producing after 48 hrs of incubation at 37°C. Moreover, in the present study 7 (32%) were found as strong biofilm forming and 15 (68%) isolates were moderate biofilm forming. None of the isolates were biofilm-negative (Figure 2). Twenty-seven percent (27%) strains were found to harbour* nuc *gene (Figure 3).* mecA* and* pvl* genes were detected in 17 out of 22 (68%) and 2 out of 22 isolates (9%), respectively. All the* pvl* harbouring isolates possessed* mec*A gene (Figure 3). The low ∆Ct value (0.493) in qRT-PCR was indicative of high expression of* pvl* in one* S. aureus *strain (Figure 4).

## 4. Discussion

In the present study, very high rates of resistance particularly towards penicillin and also for kanamycin, cefoxitin, novobiocin, and erythromycin were observed in the isolated community acquired* S. aureus* strains. In 2005, Kazakova et al. [26] reported a community-associated MRSA (CA-MRSA) clone isolated from US football players with skin abscesses. The strain was susceptible to most antimicrobial agents except *β*-lactams and macrolides. Bhatta et al. [27] reported the detection of* mecA* gene in 56.8% of the isolates from clinical and community settings. In this study, out of twenty-two clinical isolates, fifteen (68%) isolates turned out to be slime producer. Jain and Agarwal [28] reported a higher rate where 64% of staphylococcal intravascular isolates were biofilm producers by CRA method. Zalipour et al. [29] reported that forty-three (54.4%) biofilm forming strains were found in clinical specimens in Iran by CRA assay. In the current study, seven (32%) isolates were strong biofilm formers and fifteen (68%) isolates were moderate biofilm formers. Mathur et al. [30] reported that 57.8% of staphylococcal clinical isolates established biofilm-positive characteristics, and 14.47% and 39.4% exhibited high and moderate biofilm production, respectively, in India. Global emergence of MRSA is serious public health problem and challenge to clinicians. Various factors devote to drug resistance and the pathogenicity of* S. aureus*. The first PVL positive MRSA was noticed in the late 1990s and these strains got scattered worldwide in recent years [31]. The role of PVL in boosting virulence of* S. aureus* and their pathogenicity is being deliberated. Panton-Valentine leukocidin rises the pathogenicity of* S. aureus* by necrosis, quickening apoptosis and damage of polymorphonuclear and mononuclear cells, thereby contributing to mortality and morbidity [32]. PVL is generally used as a marker for community acquired MRSA, liable for deep dermal infections including soft tissue [33, 34]. However, the worldwide scheme of PVL among MRSA isolates varies. A lower prevalence of PVL has been reported from other parts of world (5% in France, 4.9% in UK, 8.1% in Saudi Arabia, and 14.3% in Bangladesh) [32, 35–37] reflecting the significant variation in prevalence of PVL among geographical areas and communities. Kaur et al. [38], from India, have reported overall 62.85% prevalence of PVL among MRSA and MSSA (MRSA: 85.1% and MSSA: 48.8%), which delineates higher prevalence of PVL among MRSA in comparison to present findings. Johnsson et al. [39] detected* pvl *gene in one isolate (1%) from 65 patients with* S. aureus *bacteraemia, in two (2.19%) isolates from 91 patients with cutaneous infections, and in four (7.27%) isolates from 55 patients with respiratory tract infections. Rostamzad et al. [40] reported that out of thirty-two MRSA isolates, thirteen isolates (40.62%) were positive for presence of the* luk-pv* gene from hospitals isolates. Higher prevalence of PVL among children (<14 years of age) was observed as compared to adults and old age group patients. Similar observations were made in another study from India [41]. Epidemiological data suggest that high virulence of community acquired MRSA is associated with* pvl* gene but direct evidence of association of PVL to pathogenesis remains limited [42]. The low ∆Ct value (0.493) in qRT-PCR indicated high expression of* pvl *gene in* S. aureus *strain in the present study. However, in previous study low expression of* luk-pv* was reported [43]. In* S. aureus, *expression of toxins and others factors can be measured by using Panton-Valentine leukocidin (PVL) expression in* in vivo* condition [44]. In addition, expression of* pvl *gene is enhanced by sub-MIC values of *β*-lactam antibiotics [45]. The studied population has a little health education and they commonly use *β*-lactam antibiotics to treat any kind of infections; this may be the reason of high expression of* pvl *gene in* in vitro* condition. Thus, detection of* pvl *gene in community isolated* S. aureus *is indication of emergence of pathogenic* S. aureus* in community setup in the studied region.

## 5. Conclusion

The current study reflects the elevated level of multi-drug resistant* S. aureus *infections in community. The prevalence of the* pvl* gene among the MRSA isolates in this study was low. The presence of* pvl* among multi-drug resistant bacteria like MRSA may be fatal and challenging condition for clinicians. The studied population has a little health education and they commonly use *β*-lactam antibiotics; this may be the underlying reason for high expression of* pvl*. Thus, detection of* pvl* in community isolated* S. aureus* indicates the emergence of pathogenic* S. aureus* in community setup in the studied region.

## Figures and Tables

**Table 1:** Source data of collected samples according to collection site, sex, and age groups of infected individual.

Superficial infection site (n=5)				Nasal swab (n=20)			
Male (n=3)		Female (n=2)		Male (n=7)		Female (n=13)	
≤30 years	≥31 years	≤30 years	≥31 years	≤30 years	≥31 year*s*	≤30 year*s*	≥31 year*s*
2	1	1	1	4	3	8	5

**Table 2:** Biochemical identification and characterization of *S. aureus*.

Tests	Source of the isolates		Total
	Superficial infection site	Nasal swab	
	No*∗* (%)	No*∗* (%)	No*∗* (%)
Colony pigment			
White	01(20)	2 (11.8)	3 (14)
Creamy	1 (20)	3 (17.6)	4 (18)
Yellow	3 (60)	12 (70.6)	15 (68)
Gram Stain	5 (100)	17 (100)	22 (100)
Catalase activity	5 (100)	17 (100)	22 (100)
Coagulase activity	5 (100)	15 (88)	20 (91)
Tnase activity	4 (80)	1 (06)	5 (23)
Mannitol fermentation with high salt concentration	5 (100)	17 (100)	22 (100)
Gelatinase activity	4 (80 )	16 (94)	20 (91)
Protease activity	3 (60 )	12 (71)	15 (68)
Urease activity	4 (80 )	15 (88)	19 (86)
Lipase production	2 ( 40)	10 (59)	12 (55)
Esculin hydrolysis	1 (10)	5 (29)	5 (27)

*∗* Positive number, percentage is presented in parenthesis.

**Table 3:** Primer sequence of *16S rRNA, mecA, nuc, pvl, *and *recA* genes of *S. aureus*.

**Target gene**	**Direction**	**Primer sequence**	**Amplicon size (bp)**	**References**
***16S rRNA***	F R	GTAGGTGGCAAGCGTTATCC CGCACATCAGCGTCAG	228	[9]
***nuc***	F R	GCGATTGATGGTGATACGGTT AGCCAAGCCTTGACGAACTAAAGC	270	[10]
***mecA***	F R	GTGAAGATATACCAAGTGATT ATGCGCTATAGATTGAAAGGAT	147	[11]
***pvl***	F R	ATCATTAGGTAAAATGTCTGGACATGATCCA GCATCAAGTGTATTGGATAGCAAAAGC	433	[12]
***recA***	F R	AAAGTTCAGGTAAGACGACAG TCCCATTTCACCTTCAATTTCAG	277	[13]

## Data Availability

The data used to support the findings of this study are available from the corresponding author upon request.
